# The Effect of Exogenous Zinc Concentration on the Responsiveness of MC3T3-E1 Pre-Osteoblasts to Surface Microtopography: Part II (Differentiation)

**DOI:** 10.3390/ma7021097

**Published:** 2014-02-11

**Authors:** Kathryn Dorst, Derek Rammelkamp, Michael Hadjiargyrou, Yizhi Meng

**Affiliations:** 1 Department of Materials Science and Engineering, Stony Brook University, Stony Brook, NY 11794-2275, USA; E-Mails: kathryn.dorst@stonybrook.edu (K.D.); derek.rammelkamp@stonybrook.edu (D.R.); 2 Department of Life Sciences, New York Institute of Technology, Old Westbury, NY 11568-8000, USA; E-Mail: mhadji@nyit.edu; 3 Department of Chemical and Molecular Engineering, Stony Brook University, Stony Brook, NY 11794-2275, USA

**Keywords:** osteoblast, zinc, micropatterns, transforming growth factor-beta 1, metalloproteinase-2

## Abstract

Osseointegration of bone implants is a vital part of the recovery process. Numerous studies have shown that micropatterned geometries can promote cell-substrate associations and strengthen the bond between tissue and the implanted material. As demonstrated previously, exogenous zinc levels can influence the responsiveness of pre-osteoblasts to micropatterns and modify their migratory behavior. In this study, we sought to determine the effect of exogenous zinc on differentiation of osteoblasts cultured on micropatterned *vs.* planar substrates. Levels of activated metalloproteinase-2 (MMP-2) and transforming growth factor-beta 1 (TGF-β1), as well as early stage differentiation marker alkaline phosphatase, were altered with the addition of zinc. These results suggest that exogenous zinc concentration and micropatterning may interdependently modulate osteoblast differentiation.

## 1. Introduction

Bone tissue morphogenesis is an intricately regulated process that depends on the interplay between cells and their extracellular matrix (ECM). These interactions can be elucidated by utilizing ECM-mimicking substrates *in vitro* [1]. Micro-topographies have been determined to be an important factor for guiding cell adhesion and enhancing vital *in vivo* functions [2,3]. Studies demonstrate that polymeric microscale ridges/grooves promote the adhesion and differentiation of human osteoblasts [3–5]. The presence of these surface features has also been shown to induce the expression of bone-specific proteins such as osteocalcin and osteopontin, even in the absence of osteoinductive media [6,7]. Additionally, surface microtopography can modulate the extent to which the cell body is elongated, which has been demonstrated to be important in determining the osteogenic capacity of osteoblasts. For example, Jansen *et al*. [8] observed that polarization of human fetal pre-osteoblasts reduced the activity of the early stage differentiation marker, alkaline phosphatase (ALP), but increased calcium deposition. Kim *et al.* [9] observed that polarizing stretch of human mesenchymal stem cells increased osteopontin but decreased osteocalcin gene expression. These studies highlight the complex role that substrate surface topography plays in directing mesenchymal cell fate [10,11].

It has also been shown that micro-textured surfaces can enhance cell responsivity to nutritional co-factors [12,13]. Zinc, which is present in the active site of ALP, is involved in the production of inorganic phosphate [14] and the calcification of the ECM [15]. Depriving pre-osteoblasts of zinc reduces their differentiation capacity by decreasing ALP and osteopontin expression, as well as calcium deposition [14]. We have previously demonstrated that levels of exogenous zinc can disrupt the interaction of pre-osteoblasts with topographical surface features, particularly in their actin organization and contact guidance behavior [16]. Because contact guidance is closely linked to osteogenic differentiation and mineralization [17–19], zinc may also have an effect on the differentiation of osteoblasts cultured on various topographies.

Many factors vital to osteoblast differentiation and ECM remodeling are zinc dependent, such as metalloproteinase-2 (MMP-2). Secreted in its inactive form, a cysteine-rich pro-domain prevents hydrolysis of the zinc binding site until phosphorylation by ALP [20,21]. The activated form of MMP-2 stimulates ECM turnover, which allows for the multi-layering of cells that is required for large bone nodule formation [20]. Blocking the activation of MMP-2 results in bone defects and delayed mineralization of the ECM [22,23]. MMP-2 activates the cytokine transforming growth factor-beta 1 (TGF-β1) [24], which is found in high quantities in bone and plays a role in a number of diverse biological processes such as cell migration, proliferation and differentiation [24,25]. In particular, TGF-β1 is especially important in regulating bone formation and is a known chemoattractant for osteoblasts [24–26]. In the absence of TGF-β1, osteoblast proliferation, matrix deposition, and collagen maturity are severely diminished [24,27,28]. Increased levels of zinc have been shown to stimulate TGF-β1 production and activation in rats [29]. The highest TGF-β1 activation levels occur during the osteoblast maturation period (10–16 days after exposure to induction media), which coincides with the production of collagen I and alkaline phosphatase (ALP). TGF-β1 levels naturally dwindle during the mineralization period (16–28 days) [26,30,31], and exogenous addition of TGF-β1 can decrease production of osteocalcin and ALP, thus prohibiting bone formation [32–34].

It has been demonstrated that both MMP production and TGF-β1 activation can be altered by surface microtopography [35,36] or by increasing the exogenous zinc concentration [37]. Therefore in our current study, we hypothesized that increasing exogenous zinc concentration would alter MMP-2 and TGF-β1 production, and subsequently differentiation, of osteoblasts cultured on various surface dimensions. To this end, we fabricated PDMS micropatterns as previously described [16], that contained either wide (20 μm width, 30 μm pitch, 2 μm height) or narrow (2 μm width, 10 μm pitch, 2 μm height) linear ridges. Flat PDMS and tissue culture polystyrene (TC) were used as the planar controls. The MC3T3-E1 subclone 4 cell line was then cultured on these surfaces for up to 28 days in osteogenic induction medium.

## 2. Results and Discussion

### 2.1. Results

#### 2.1.1. Metalloproteinase-2 (MMP-2)

**Figure 1.**
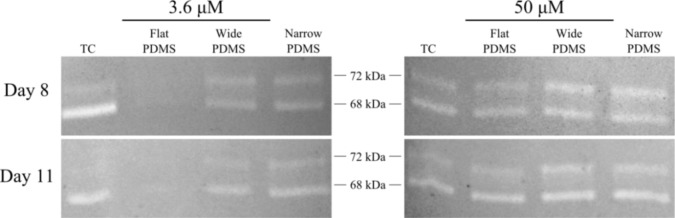
Gelatin zymogram demonstrating MMP-2 activity (indicated by white bands). Subclone 4 MC3T3-E1 cell extracts on PDMS and tissue culture polystyrene (TC) surfaces in serum level zinc (3.6 μM) and zinc-rich (50 μM) media conditions were isolated on day 8 and 11, and normalized to a total protein concentration of 17.90 μg per well. Zymograms shown are representative of data from two independent experiments.

**Figure 2.**
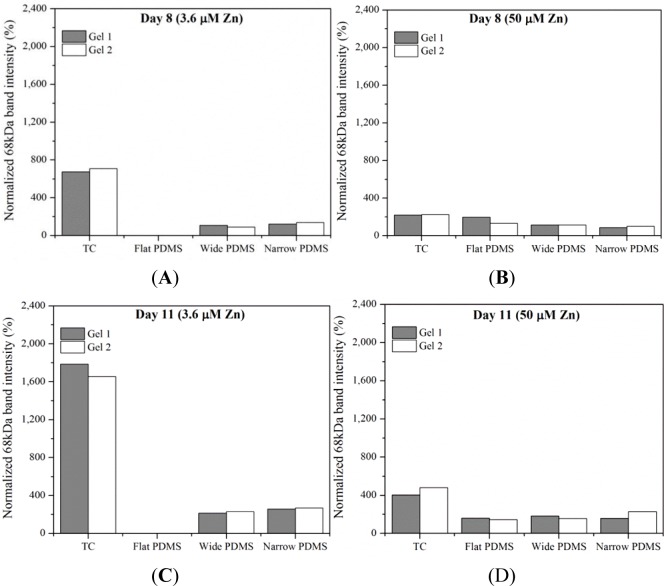
Intensity of 68 kDa gel zymogram bands (normalized to the 72 kDa bands of each corresponding gel) from extracts of MC3T3-E1 cells collected on day 8 in (**A**) 3.6 μM Zn and (**B**) 50 μM Zn, and on day 11 in (**C**) 3.6 μM Zn and (**D**) 50 μM Zn. Data represent band intensities from two independent gel zymography experiments. Intensity values were not reported for extracts from flat PDMS samples at 3.6 μM zinc because the bands were too faint.

Results from gelatin zymography showed the presence of both latent pro-MMP-2 (72 kDa) and the active form of MMP-2 (68 kDa) for some, but not all, of the cell extracts collected on days 8 and 11 (Figure 1). At serum-level (3.6 μM) zinc, total MMP-2 production on either day was highest in cells cultured on TC and patterned PDMS, as can be seen by clear, white bands at 72 kDa and 68 kDa (Figure 1). The dual bands present in the lanes containing cell lysates from TC are very similar to those reported for human osteoblasts in the literature [38]. The relative intensity of the 68 kDa activated MMP-2 band in the TC lane is noticeably stronger than the 72 kDa band (675%–1790% higher than the 72 kDa band) at both days in serum-level zinc (Figures 1 and 2A,C). At elevated zinc concentration (50 μM), the relative intensity of the 68 kDa band from the TC samples was not as pronounced (220%–400%) (Figure 2B,D). On flat PDMS, total MMP-2 production at 3.6 μM zinc was extremely low (very faints bands in Figure 1), and the intensity of the zymogram bands was not calculated (Figure 2A,C). When zinc was elevated to 50 μM, MMP-2 production was much more noticeable (Figure 1) and the intensity of the 68 kDa band was calculated to be similar to that of the 72 kDa band (160%–200%, Figure 2B,D). On patterned PDMS, the relative intensities of the 68 kDa band from day 8 lysates remained unchanged when zinc concentration was increased (110%–125% overall increase) (Figure 2A,B). The relative intensities of the day 11 lysates were higher (160%–260%) but also did not vary with zinc concentration (Figure 2C,D).

#### 2.1.2. Transforming Growth Factor-Beta 1 (TGF-β1)

**Figure 3.**
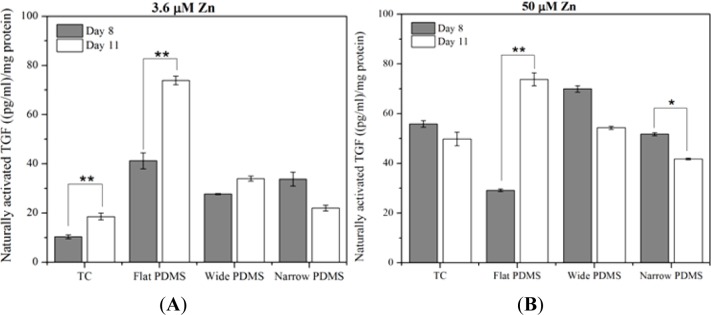
TGF-β1 activation for subclone 4 MC3T3-E1 cells cultured for 8 and 11 in: (**A**) 3.6 μM Zn media and (**B**) 50 μM Zn media, normalized to total protein. A single asterisk (*) indicates a *p* < 0.01 level of significance while double asterisks (**) indicate a *p* < 0.05 level of significance.

At serum-level zinc, TGF-β1 activation in MC3T3-E1 cells cultured on TC was lowest but rose significantly (*p* = 0.035), doubling from day 8 to day 11 (Figure 3A), similar to temporal expression data reported in the literature [39]. On flat PDMS, activation levels were the highest and there was a significant increase with time (78%; *p* = 0.012). On the patterned PDMS, TGF-β1 activation levels were much lower compared to flat PDMS and either slightly increased (21%, wide) or decreased (33%, narrow) from day 8 to day 11 (Figure 3A).

At 50 μM zinc, TGF-β1 activation for cells on TC did not vary significantly (*p* = 0.296) between days 8 and 11 (Figure 3B). Activation on flat PDMS continued to rise significantly (*p* = 0.038) with incubation time and showed a 150% increase from day 8 to day 11. On patterned PDMS there was a slight decrease (23%) in activation for the cells on wide patterns (*p* = 0.057) and a significant decrease (20%) for those on narrow patterns (*p* = 0.005).

Increasing the exogenous zinc concentration from 3.6 μM to 50 μM resulted in a significant increase in TGF-β1 activation that was only seen in MC3T3-E1 cells cultured on either TC for 8 days (450%; *p* = 0.001) or on wide patterned PDMS for 11 days (160%; *p* = 0.003). In contrast, the level of activated TGF-β1 in cells cultured on flat PDMS was not responsive to zinc and either slightly decreased (30% on day 8; *p* = 0.169) or remained unaltered (day 11; Figure 3A,B).

#### 2.1.3. Alkaline Phosphatase (ALP)

**Figure 4.**
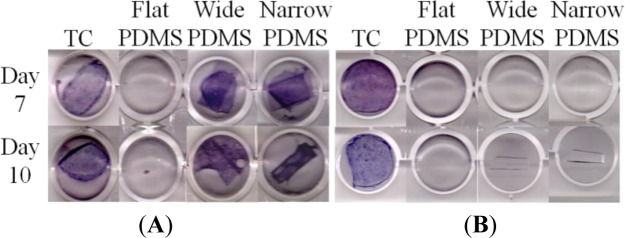
Alkaline phosphatase (ALP) production (indicated by dark purple color) by subclone 4 MC3T3-E1 cells in: (**A**) 3.6 μM zinc and (**B**) 50 μM zinc. Micrographs are representative of data collected from two independent experiments.

On tissue culture, ALP production was strongly positive for MC3T3-E1 cells cultured for 7 or 10 days in induction medium at both serum-level (Figure 4A) and elevated (Figure 4B) zinc concentrations, as indicated by the dark purple coloration in the tissue culture well. This was expected of the subclone 4 of the MC3T3-E1 cell line, whose ALP production typically peaks around 14 days [40–42]. For cells that were cultured on flat PDMS, ALP activity was consistently negative irrespective of zinc concentration. On the patterned PDMS surfaces, ALP was positive only at serum-level zinc (3.6 μM, Figure 4A), and was negative at elevated zinc (50 μM, Figure 4B).

#### 2.1.4. Von Kossa

**Figure 5.**
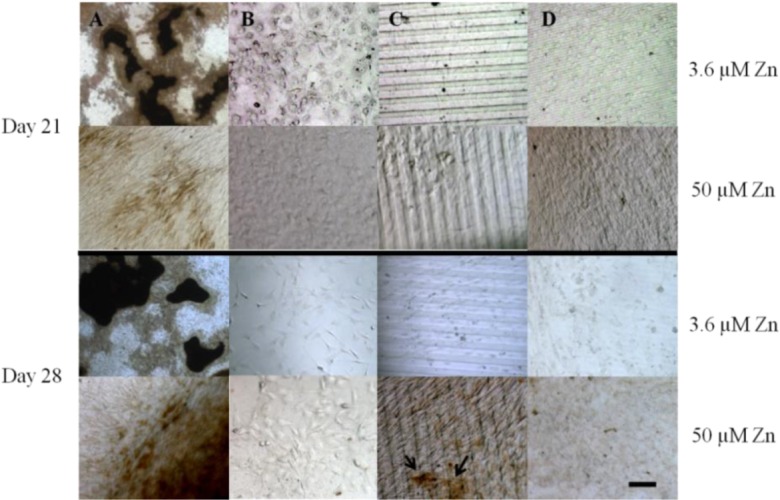
Von Kossa staining for calcium phosphate bone nodules (indicated by black areas) of subclone 4 MC3T3-E1 cells on: (**A**) TC; (**B**) flat PDMS; (**C**) wide PDMS ridges; (**D**) narrow PDMS ridges. Areas are representative of up to six fields of view collected from three independent experiments. Bar = 100 μm.

**Figure 6.**
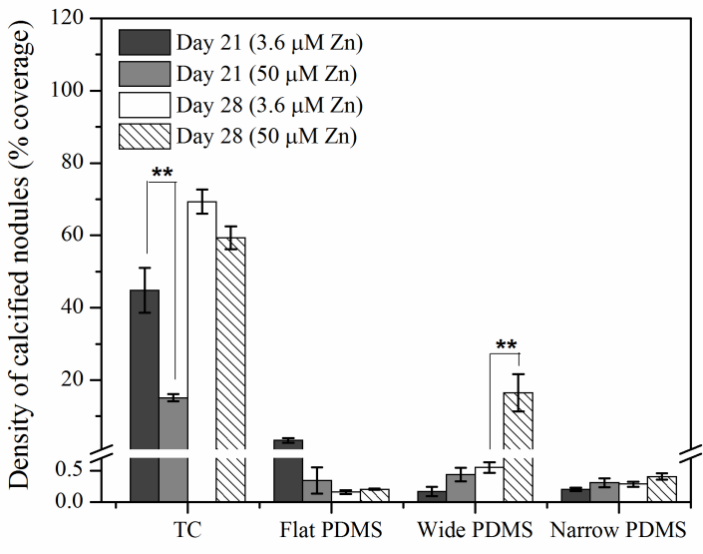
Quantitative calcified nodule density in MC3T3-E1 cell cultures after 21 and 28 days of exposure to induction medium. Densities represent the average area coverage of up to six fields of view collected from three independent experiments. Double asterisks (**) indicate a *p* < 0.05 level of significance.

Von Kossa staining for calcium deposition revealed an abundance of dark, punctate nodules in MC3T3-E1 cells cultured on TC after 21 days at serum-level zinc (3.6 μM; Figures 5A and 6), similar to previous reports for this cell line [40]. At elevated levels of zinc (50 μM), the von Kossa staining was lighter and more diffuse in color, and the area coverage of calcified nodules was significantly reduced (67% decrease, *p* = 0.017) (Figures 5A and 6). On either flat (Figures 5B and 6) or narrow (Figures 5D and 6) PDMS, calcium deposition appeared to be absent regardless of zinc concentration. On the wide PDMS patterns (Figure 5C), nodules were absent at serum-level zinc but small and sparsely scattered clusters began to be visible on day 28 at elevated zinc (Figure 6 and arrows in Figure 5C). Area coverage of calcified nodules also increased significantly compared to serum-level zinc (*p* = 0.019).

### 2.2. Discussion

Cell differentiation is a complex process that requires the precise timing and convergence of physical and biochemical factors in the micro-environment of the extracellular matrix (ECM) [2,43]. These factors can be modulated by micro- and nano-topographical features of the substrate and can induce the maturation of pre-osteoblasts into mineralized tissue [12,44,45]. We have previously shown that exogenous zinc can disrupt the directional migration of MC3T3-E1 pre-osteoblasts cultured on anisotropic PDMS surfaces patterned with 2 μm or 20 μm wide ridges [16]. Here we sought to investigate the effect of exogenous zinc on the differentiation of MC3T3-E1 cells cultured on micropatterns of the same dimensions.

Zinc is a crucial trace metal for regulating bone homeostasis [14,46–49]. For example, the Zip13 and Zip14 zinc transporter proteins have been shown to be factors in the coordination of mammalian cell growth through BMP/TGF-β1-mediated signaling pathways [50,51]. Exogenous zinc levels have been shown to directly stimulate the production of TGF-β1 in newborn rats [29], and the precisely-timed activation of TGF-β1 is critical in the osteoblast mineralization process [27,28,34]. Latent TGF-β1 contains a metalloproteinase cleavage site for activation [24], upon which local ECM remodeling is stimulated and bone formation is induced [52]. Moreover, it has been shown that MMP-2 is directly involved in orthopedic wound healing *in vivo* and is associated with bone turnover and bone formation [53]. Secreted in its latent form, as pro-MMP-2, it is preferentially activated by a cell membrane-tethered MMP, MT1-MMP, which is involved in ECM turnover and is essential for osteoblast multi-cell layering during ECM mineralization [20,54,55].

Based on these previous studies, we first examined the activity levels of MMP-2 and TGF-β1 in extracts of MC3T3-E1 cells cultured on planar and micropatterned PDMS. We observed that at serum-level (3.6 μM) zinc, total MMP-2 production was much greater in osteoblasts cultured on patterned PDMS containing 2 μm and 20 μm wide ridges than on the flat PDMS, indicating that ECM remodeling was more extensive [36]. When exogenous zinc concentration was increased to 50 μM, relatively little difference in MMP-2 activity was seen in the cells on patterned PDMS, however dramatic increases were observed in the cells on flat PDMS. This indicates that elevating the zinc concentration may not have an effect if the ECM remodeling process is already under way, such as on the patterned PDMS substrates.

Here we also observed that levels of activated TGF-β1 in MC3T3-E1 cells cultured on patterned PDMS were increased at elevated levels of exogenous zinc. On the contrary, levels of activated TGF-β1 in cells cultured on flat PDMS did not respond to higher zinc concentrations and continued to increase rapidly from day 8 to day 11, indicating that a peak maximum had not yet been reached. This suggests that TGF-β1 activation on planar and microtopographied surfaces has distinctly different temporal profiles, perhaps partly due to differences in the stability of MMP-2 activity levels. These observations are in agreement with previous reports that showed the stimulatory effect of zinc and patterning on TGF-β1 secretion [29,56].

We next assessed alkaline phosphatase (ALP) activity of the MC3T3-E1 cells to evaluate their early stage osteogenic capacity. Using a histological staining assay [40,57,58], we observed that ALP staining on TC was strongly positive at both serum-level and elevated zinc concentrations, as expected [14,59,60]. ALP activity for MC3T3-E1 cells on flat PDMS remained low and did not vary with increased zinc concentration, which was not surprising because TGF-β1 levels were also not responsive to levels of exogenous zinc. At serum-level zinc, ALP activity on the micro-patterned PDMS was much higher than on flat PDMS, similar to a recent study of MC3T3 cells on micropatterned titanium [61]. This was expected from the gel zymography data, as it has been suggested that the activity of MMP-2 can be modulated by ALP via dephosphorylation [21]. It is also possible that the presence of micropatterns helped to stabilize the levels of activated TGF-β1, similar to that on TC, and as a result promoted early stage differentiation. It was surprising to find that, at elevated zinc concentration, ALP activity was greatly reduced on both patterned PDMS substrates. We believe that this loss may be related to decreased MMP-2 activity at 50 μM zinc that is reflected by a drop in the intensity of the activated 68 kDa zymogram band (Figure 2). This indicates a concurrent decrease in the activity of MT1-MMP, which has been shown to regulate ALP expression [62]. Consequently, ALP activation is attenuated. Another possibility is that exogenous zinc transported inside the cell by channels/transporter proteins directly interfered with the synthesis of ALP, leading to reduced activation of MMP-2 and TGF-β1 [21,59].

Results from the von Kossa staining assays showed that MC3T3-E1 cells cultured on TC calcified extensively after 21 days of exposure to induction medium at serum-level zinc. Calcification was much less extensive at elevated zinc, which is similar to previous findings [14,40,42,60]. Calcified nodules were clearly absent in all cell cultures on flat PDMS, irrespective of zinc concentration. This is in alignment with their low ALP activity. It is possible that elevated levels of activated TGF-β1 in the ECM may have interrupted the differentiation process. TGF-β1 is known to have a paradoxical role during bone formation, as it stimulates bone formation but can also inhibit mineralization if excess amounts remaining in the matrix are not inactivated or removed [24,39,63,64]. Although ALP activity was high in cells cultured on patterned PDMS at serum-level zinc, calcification did not occur. This suggests that while levels of inorganic phosphate may be high, calcium levels in the matrix may have been diminished, possibly due to competition between Ca and Zn [65]. Low extent of calcification observed in cells on the wide PDMS patterns at elevated zinc does not agree with the low ALP data and could be indicative of the limit of detection with histological assays.

## 3. Experimental Section

### 3.1. Substrate Fabrication

The creation of micropatterns in PDMS was described previously [16]. Briefly, standard photolithography was used to create sections of negative resist from which PDMS replicas containing two patterns (20 μm wide with a 30 μm pitch and 2 μm height, and 2 μm wide with a 10 μm pitch and 2 μm height) were created. PDMS substrates were rendered hydrophilic using plasma oxygen [66]. As a control, unaltered 24-well tissue culture (TC) plates (BD Falcon, Bedford, MA, USA) were used. PDMS substrates were functionalized with fibronectin, at 10 μg/mL (Sigma Aldrich, St. Louis, MO, USA) followed by bovine serum albumin (EMD Chemicals; Gibbstown, NJ) to block non-specific protein binding.

### 3.2. Cell culture and Development

Pre-osteoblast MC3T3-E1 cells (subclone 4; American Type Culture Collection (ATCC), Manassas, VA, USA) were maintained using MEM-α (Gibco/Invitrogen, Grand Island, NY, USA) supplemented with 10% fetal bovine serum (FBS; Hyclone/Thermo Fisher Scientific; Logan, UT, USA) and 1% penicillin-streptomycin (Gibco/Invitrogen) at 37°C with 5% CO_2_, 95% relative humidity. Media was supplemented with 4 mM glycerol 2-phosphate (Sigma Aldrich) and 50 μg/mL sodium L-ascorbate (Sigma Aldrich) and replaced every 2–3 days. Subclone 4 MC3T3-E1 pre-osteoblasts were chosen due to their demonstrated ability to produce copious amounts of calcium phosphate nodules on TC [40].

As described previously [16], zinc levels were varied using a standardized process by Prasad *et al*. [67] and serum levels of zinc were reported as 3.6 μM [68]. A zinc-rich condition of 50 μM was used to mimic zinc supplementation to a deficient system.

### 3.3. MMP-2 Activity: Gelatin Zymography

Twenty-four hours after seeding at 50,000 cells/cm^2^, cells were given induction medium (supplemented with glycerol 2-phosphate and sodium L-ascorbate) for a period through 11 days. At each time point, the samples were serum starved for 24 h before supernatant was removed from the cell sample and lysis buffer (Tris-HCl, Triton X-100) was added and the plate frozen at −80°C. Samples were taken through several freeze-thaw cycles to release the cell layer from the sample. Additional cell layer removal was done by scraping and the lysates were then transferred to individual tubes. Next they were passed through a needle several times before adding to a QIAShredder (Qiagen, Hilden, Germany) containing a filter and a collection tube and then centrifuged to remove any cell structural components.

Using a Nanodrop-1000 spectrophotometer (Nanodrop Technologies, Wilmington, DE, USA) samples were normalized to protein concentration and added 1:1 with Tris-Glycine SDS sample buffer (Tris-HCl, glycerol, SDS, bromophenol blue, DI water) before run on the 10% pre-made gel zymography gel (Biorad, Hercules, CA, USA). Tris-Glycine SDS running buffer (Tris base, glycine, SDS, DI water) was used as reservoir buffer in the gel box and a constant voltage of 125 V was maintained. Subsequent to running, samples were then incubated for 30 min in zymogram renaturing buffer consisting of 2.5% (v/v) Triton-X-100. The renaturing buffer was then decanted and developing buffer (Tris base, Tris-HCl, NaCl, CaCl_2_, Brij 35, DI water) was added to the gel for 30 min. The developing buffer was then replaced with fresh developing buffer and incubated with the gel at 37°C overnight. The developing buffer was then removed and a Coomassie blue stain was incubated with the gel at room temperature for 30 min and subsequently decanted and rinsed with destaining solution over several hours until bands were clear. Areas of protease activity appeared as clear bands against a dark blue background where the protease has digested the substrate. Images were captured using a color photo scanner. The intensity of 72 kDa and 68 kDa bands were calculated using ImageJ (NIH) after the photograph was first converted to a binary image and thresholded. The intensities of the 68 kDa band were normalized to that of the 72 kDa band in each corresponding gel. The experiment was conducted two times and zymography data from both gels were analyzed.

### 3.4. TGF-β1 ELISA

Cell lysates were prepared similarly to that of the MMP-2 samples. Briefly, cells were seeded at 50,000 cells/cm^2^ and given induction medium (supplemented with glycerol 2-phosphate and sodium L-ascorbate) for 8 and 11 days and serum-starved for 24 h before adding lysis buffer and freezing at −80°C. Additional cell layer removal was done by scraping and the lysates were then transferred to individual tubes. Next they were passed through a needle several times before adding to a QIAShredder containing a filter and a collection tube and then centrifuged to remove any cell structural components. Using a Nanodrop-1000 spectrophotometer samples were analyzed for protein concentration.

Activated TGF-β1 was determined using the TGF-β1 E_max_ ImmunoAssay System (Promega Corp., Madison, WI, USA). The assay was performed according to the manufacturer’s directions. Briefly, samples were diluted with PBS to an appropriate range for *in vitro* cell culture samples and split into two sets. The first set was diluted again to the final concentration for use, these samples contained only naturally processed TGF-β1 and served as the representation of amount of activated TGF-β1. The second set was acid treated with hydrochloric acid and then neutralized with sodium hydroxide to activate all the TGF-β1 in the samples, serving as representation of total amount of both activated and latent TGF-β1. Samples were normalized to average mass of total protein amount.

### 3.5. Alkaline Phosphatase Production (ALP)

Twenty-four hours after seeding at 50,000 cells/cm^2^, cells were given induction medium (supplemented with glycerol 2-phosphate and sodium L-ascorbate) for 7 days or 10 days. At each time point, an ALP substrate buffer was prepared using 2-amino-2-(hydroxymethyl)-1,3-propanediol (Tris base, Applied Science, Indianapolis, IN, USA), sodium chloride (Sigma-Aldrich), and magnesium chloride (Sigma-Aldrich), with the pH adjusted to 9.5 using hydrochloric acid. Samples were rinsed with Tris-Buffered Saline Tween-20 (TBST) before starting the assay. A working reagent of nitro blue tetrazolium (NBT, Sigma Aldrich) and 5-bromo-4-chloro-3-indolyl phosphate (BCIP, Sigma Aldrich) was prepared fresh and added to each sample and allowed to incubate for 10 min at room temperature. Color development was stopped by rinsing the samples with deionized water. Images were captured using a color photo scanner. Micrographs are representative of data collected from two independent experiments.

### 3.6. Calcium Phosphate Nodules (Von Kossa)

Cells used for von Kossa samples were cultured in induction medium for 21 and 28 days after seeding at 50,000 cells/cm^2^. At each time point the samples were rinsed twice with PBS and fixed using formaldehyde in PBS for 10 min. Following a water rinse, the cells were covered with a silver nitrate solution and exposed to UV light for 20 min. The samples were then rinsed with water and covered with sodium thiosulfate (Electron Microscopy Sciences, EMD, Hatfield, PA, USA) for 3 min, followed by final water rinse. A standard inverted microscope with a color camera was used to capture images in a range of magnifications. Micrographs were collected from three independent experiments (up to 6 images per experiment), and the area coverage of calcified nodules at the lowest magnification (2.5×) was quantified by converting images to binary and thresholding using ImageJ.

### 3.7. Statistics

Each experiment consisted of a minimum of three replicates per treatment group. Statistical analysis was performed using an unpaired student *t*-test to and differences were considered significant at the level of *p* = 0.05.

## 4. Conclusions

In this study, we observed that differentiating MC3T3-E1 osteoblasts cultured on patterned PDMS are more responsive to changes in the level of exogenous zinc than osteoblasts cultured on flat PDMS. Consequently, TGF-β1 activation is more stable and early bone differentiation (alkaline phosphatase) is enhanced. These differences may be explained by the increased clustering of actin fibers on the micropatterns, which may increase cytoskeletal tension that, in turn, promotes osteoblast differentiation. Taken together with our previous study on contact guidance, these results collectively suggest that anisotropic migration and exogenous zinc concentration may interdependently modulate osteoblast differentiation.
